# Development of an explainable AI system using routine clinical parameters for rapid differentiation of inflammatory conditions

**DOI:** 10.3389/fimmu.2024.1364954

**Published:** 2024-03-06

**Authors:** Joerg Hoffmann, Anne Rheude, Andreas Neubauer, Cornelia Brendel, Michael C. Thrun

**Affiliations:** ^1^ Department of Hematology, Oncology and Immunology, Philipps University Marburg, University Hospital Giessen and Marburg, Marburg, Germany; ^2^ Databionics, Mathematics and Computer Science, Philipps University Marburg, Marburg, Germany

**Keywords:** explainable AI, inflammation, CD64, CD169, infection

## Abstract

**Introduction:**

Inflammatory conditions in patients have various causes and require different treatments. Bacterial infections are treated with antibiotics, while these medications are ineffective against viral infections. Autoimmune diseases and graft-versus-host disease (GVHD) after allogeneic stem cell transplantation, require immunosuppressive therapies such as glucocorticoids, which may be contraindicated in other inflammatory states. In this study, we employ a combination of straightforward blood tests to devise an explainable artificial intelligence (XAI) for distinguishing between bacterial infections, viral infections, and autoimmune diseases/graft-versus-host disease.

**Patients and methods:**

We analysed peripheral blood from 80 patients with inflammatory conditions and 38 controls. Complete blood count, CRP analysis, and a rapid flow cytometric test for myeloid activation markers CD169, CD64, and HLA-DR were utilized. A two-step XAI distinguished firstly with C5.0 rules pruned by ABC analysis between controls and inflammatory conditions and secondly between the types of inflammatory conditions with a new bivariate decision tree using the Simpson impurity function.

**Results:**

Inflammatory conditions were distinguished using an XAI, achieving an overall accuracy of 81.0% (95%CI 72 – 87%). Bacterial infection (N = 30), viral infection (N = 26), and autoimmune diseases/GVHD (N = 24) were differentiated with accuracies of 90.3%, 80.0%, and 79.0%, respectively. The most critical parameter for distinguishing between controls and inflammatory conditions was the expression of CD64 on neutrophils. Monocyte count and expression of CD169 were most crucial for the classification within the inflammatory conditions.

**Conclusion:**

Treatment decisions for inflammatory conditions can be effectively guided by XAI rules, straightforward to implement and based on promptly acquired blood parameters.

## 1 Introduction

Bacterial and viral infections, often manifesting with symptoms such as respiratory distress and diarrhoea, represents some of the most common reasons for emergency department visits and hospital admissions in the United States even before the advent of COVID-19 (1). Upon presentation, these patients frequently exhibit inflammatory signs such as fever, increased respiratory rate, low blood pressure, high heart rate, and elevated inflammation markers like C-Reactive Protein (CRP) and leukocytes. Inflammation plays a pivotal role in a broad spectrum of physiological and pathological processes, including infection, tissue injury, and stress or malfunction of tissues (2).

Patients are often inaccurately treated with empirical antibiotics, particularly when presenting with respiratory tract symptoms (3–5). This approach is ineffective, especially for viral infections that do not respond to antibiotics and can even be inappropriate or contraindicated in patients with inflammatory symptoms due to autoimmune processes like vasculitis or graft-versus-host disease (GVHD) following allogeneic stem cell transplantation. These situations warrant the use of immunosuppressive treatment. Thus, developing quick, easy to assess, and reliable tests to distinguish between different sources of inflammation is imperative to aid clinicians in making appropriate treatment decisions.

Several studies have evaluated using CRP, white blood cell (WBC) count, and procalcitonin (PCT), either individually or in various combinations, as markers to differentiate between bacterial and non-bacterial infections, with inconsistent results as reviewed elsewhere (6). A meta-analysis of 14 studies, encompassing 2,471 patients, conducted by Yeh et al., demonstrated that expression of the Fc gamma receptor I (CD64) on polymorphonuclear neutrophils (PMN) was superior to CRP and PCT for predicting sepsis (7). Moreover, Hussein et al. employed CD64 expression on PMN to distinguish between infections and disease flare-ups in patients with autoimmune disease (8).

Nonetheless, interferon gamma (IFNγ), known to induce CD64 expression, plays a pivotal role in the immune regulation of viral and bacterial infections, cancer, and autoimmune diseases (9). As such, relying solely on CD64 expression may lead to misinterpretation of inflammatory states. Bourgoin et al. demonstrated that a combination of CD64 and CD169 (Siglec-1) expression on white blood cells could aid in differentiating between viral and bacterial infections (10–12). Furthermore, Rose et al. found a correlation between increased CD169 and disease activity in patients with Sjögren’s syndrome (13). HLA-DR, an MHC-Class II receptor, has been shown to decrease in sepsis-induced immunosuppression (14, 15), and is associated with poor outcomes in sepsis patients (16, 17).

In this study, we employed a rapid, no-wash flow cytometry test for measuring CD64, CD169, and HLA-DR on monocytes and PMN, integrating these results with differential blood counts and CRP values. The comprehensive data set, obtainable within two hours in a laboratory setting, was subsequently fed into an explainable artificial intelligence (XAI) system. This white box model is designed to differentiate in a manner that is understandable to physicians between bacterial, viral, and autoimmune-induced inflammation, including cases of graft-versus-host disease following allogeneic stem cell transplantation. Addressing the limitations of subsymbolic AI systems, particularly those that rely on neural networks, our XAI model provides transparent and comprehensible rationales for its decision-making process (18–20). Aligned with regulations mandating decision justification in AI systems (21), our model delivers case-specific explanations that are intelligible to physicians, thereby facilitating a more in-depth evaluation of the AI’s diagnostic reasoning.

## 2 Materials and methods

### 2.1 Patients

A total of 100 consecutive patients exhibiting inflammatory symptoms and 50 control patients without such symptoms were included in the study, following their informed consent and in accordance with the guidelines of the local ethical committee (Vote 201/20) and the Declaration of Helsinki. Patients were considered to have inflammatory symptoms if they met at least two of the following criteria, which could not be explained otherwise: temperature > 38°C or < 36°C, heart rate > 90/min, respiratory rate > 20/min, PaCO2 < 32mmHg, leukocyte count > 12,000/µL or < 4000/µL or > 10% immature granulocytes (bands), CRP > 50 mg/dL, and systolic blood pressure < 100mmHg. Additionally, we included patients with active bacterial infections, as evidenced by positive bacterial cultures, nitrite-positive urinary conditions, or characteristic pneumonia infiltrates. Patients with viral infections were confirmed through positive IgM serology, rapid antigen tests, or PCR. Those with active GVHD were identified based on histological evidence and typical clinical presentation, and patients diagnosed with autoimmune diseases were defined in accordance with the respective medical association’s criteria. Patients were assessed in two distinct cohorts. The initial cohort, designated for training and cross-validation, comprised 118 patients: 30 with bacterial infections, 26 with viral infections, 24 with autoimmune diseases/graft-versus-host disease (AID/GVHD), and 38 control patients. The second cohort, used for validation, included 32 patients: 12 with bacterial infections, 8 with viral infections, and 12 control patients. Patients with AID/GVHD were excluded from the validation cohort due to the specific focus of this phase of the study. Details regarding the diagnoses of control patients are provided in **Table S1** in the Supporting Information.

### 2.2 Collected parameters

For the study, differential blood counts were conducted using the Sysmex XS-1000i (Sysmex Corporation, Kobe, Japan). Additionally, high-sensitive CRP levels (with a detection limit of 0.20 mg/L) were measured using the SYNCHRON^®^ System (Beckman Coulter, Fullerton, CA, USA) following the manufacturer’s instructions. Furthermore, a flow cytometric analysis of monocytes and polymorphonuclear neutrophils (PMN) for CD169-PE (clone 7-239), HLA-DR-APC (clone Immu357), and CD64-PB (clone 22) (all Beckman Coulter, three marker combination, C63854) was performed on the Navios^®^ Flow Cytometer (Beckman Coulter). The relative median fluorescence intensity (MFI) for both monocytes and PMN were determined for all three antigens. Lymphocytes served as the negative population.

### 2.3 Dataset extension with synthetic patients

118 cases x 18 variables were used. We used the knn-classifier and the Bayes classifier for the baseline. We performed k-fold cross-validation with k = 30. Due to the low number of cases the dataset was extended in the next step by the SMOTE algorithm (22). The SMOTE algorithm generates synthetic examples of selected classes by creating new instances similar to the existing class samples. It does this by identifying the nearest neighbours of each class sample and creating new synthetic examples along the line segments connecting these neighbours. Depending upon the amount of over-sampling required, neighbours from nearest neighbours are randomly chosen. We set the parameter for the nearest neighbours k = 5. The data was extended by factor four. Again, we performed k-fold cross cross-validation with k = 30 with the Bayes and knn classifier to define a second baseline.

### 2.4 Construction of an explainable AI based on the diagnostic process of a physician

The XAI system was constructed in two steps, similar to the diagnostic process of a physician. In the first step, the physician decides if the patient has an inflammatory condition. In the second step, the physician decides if it is bacterial, viral, or Autoimmune disease/GVHD.

The XAI system uses in the first step k-ary C5.0 tree to distinguish between normal controls and the named diseases (23). The C5.0 decision tree is a powerful and widely used classification algorithm. It is an improved version of Quinlan’s earlier ID3 (Iterative Dichotomiser 3) (24) and C4.5 algorithms (25). C5.0 uses a divide-and-conquer approach to recursively split the data based on the most significant attributes. It selects the best attribute to split the data at each node, using criteria like information gain or gain ratio. The splitting continues until a certain stopping condition is met, such as reaching a maximum depth or the minimum number of samples per leaf. After learning the tree, we use the internal procedure of C5.0 to extract the set of rules. It should be noted that when a ruleset is used to classify a case, several of the rules may be applicable (that is, all their conditions are satisfied) (https://www.rulequest.com/see5-unix.html). Within each rule, the number of cases that satisfy these conditions are specified. This information of the number of cases is used in the ABC Analysis algorithm (26). For a given information stored as a vector, the method computes precise limits to acquire easily interpreted subsets. With the help of the ABC curve, the algorithm calculates the optimal limits by exploiting the mathematical properties pertaining to the distribution of analysed items. Closely related to the Lorenz curve, the ABC curve can visualize the data by graphically representing the cumulative distribution function. The information containing positive values is divided into three disjoint subsets A, B and C, with subset A comprising very profitable values, i.e. largest data values (“the important few”), subset B comprising values where the yield equals to the effort required to obtain it, and the subset C comprising of non-profitable values, i.e., the smallest ones. The two most important rules for class 1 are selected by this ABC analysis (26).

In the second step, a bivariate binary decision tree based on the Simpson impurity function is proposed. The term Impurity refers to the measure of the disorder in a partition of data. The impurity is used to determine the best split at each tree node during the construction process by measuring how well a split separates the classes or categories in the current partition of data (27). The aim is to minimize the impurity at each node by selecting the split that results in the purest subsets (27). This process continues recursively until a stopping criterion of having nodes with a minimum number of instances is met (27). Simpson’s impurity is a measure used in ecology to assess the diversity (28). It considers both the number of different classes (species richness) and the relative abundance of each class (evenness). It measures how evenly the cases are distributed among the different classes.

Note that by choosing decision trees to model the physician’s diagnostic process through an XAI system, the generalization ability remains unaffected through the synthetic cases (22). We performed k-fold cross-validation with k = 30.

### 2.5 Variable importance measurement

Variable importance measurement was based on the abstract of Hennig et al. (29), presented at the Joint Conference of Data Science, Statistics & Visualisation and the European Conference on Data Analysis, held in Antwerp from July 5-7, 2023, focusing on cluster analysis. After the full two-step XAI is learned, it is applied to the original dataset. For the baseline x, an adjusted rand index (30, 31) is computed using the prior classification of the dataset and the XAI predicted classification. Next, N = 200 times the values of a selected feature are randomly permutated. For each trial, the value y of the adjusted rand index is computed by using the prediction of the XAI and the prior classification. Thereafter, the relative differences (32) to the baseline are computed as follows:


R=0.5*(x−y)/(x+y)*100

The range of relative differences is the interval [-200%, 200%]. The approach yields N = 200 relative difference R values for a feature. Next, the Mirrored-density plot (MD plot) (33) estimates parameter-free the probability density function (pdf). The MD plot is a type of data visualization that displays the pdf of two or more variables in a single graph. It is often used to compare the distributions of continuous variables across different variables, groups, or conditions. The plot consists of two mirrored density curves, one for each dataset, placed on either side of a central axis. The area between the curves is filled in blue. The density curves show how the data is distributed along the range of the variable. By using MD plots, one can easily observe and compare the distributions’ shape, spread, and central tendencies between different variables. The MD plot outlines the variable importance as follows. If the blue area lies around zero with a small spread, the variable is unimportant because permutations did not influence the adjusted rand index compared to the baseline. If the area lies below zero, permutations of the variable will improve the performance compared to the baseline. If the area lies above zero, the performance of permutations of a variable is lower compared to the baseline. This indicates that the variable is important in the XAI. The variable importance is estimated with this procedure for each variable of the dataset.

## 3 Results

### 3.1 Training and cross-validation

A total of 118 patients were enrolled in the training and cross-validation cohort, categorized based on all available clinical information as follows: bacterial infections (N = 30), viral infections (N = 26), autoimmune diseases, including patients with graft-versus-host disease (N = 24), and negative controls without inflammation (N = 38). The median age of the patients was 61 years, with a range spanning from 18 to 91 years. 42% of the participants were female, and 58% were male. The age and sex of the subgroups are disaggregated in **Table 1**.

**Table 1:** Characteristics of Subgroups.

	Age in years – median (range)	Female/Male – N
Total	61 (18 - 91)	49/69
**Bacterial infection**	64.5 (18 - 91)	10/20
** Viral infection**	61 (20 - 89)	9/17
** Autoimmune** ** disease/GVHD**	61.5 (21 - 84)	14/10
** Controls**	59 (20 - 82)	16/22

N, Number; GVHD, Graft versus host disease.

**Figure 1:**
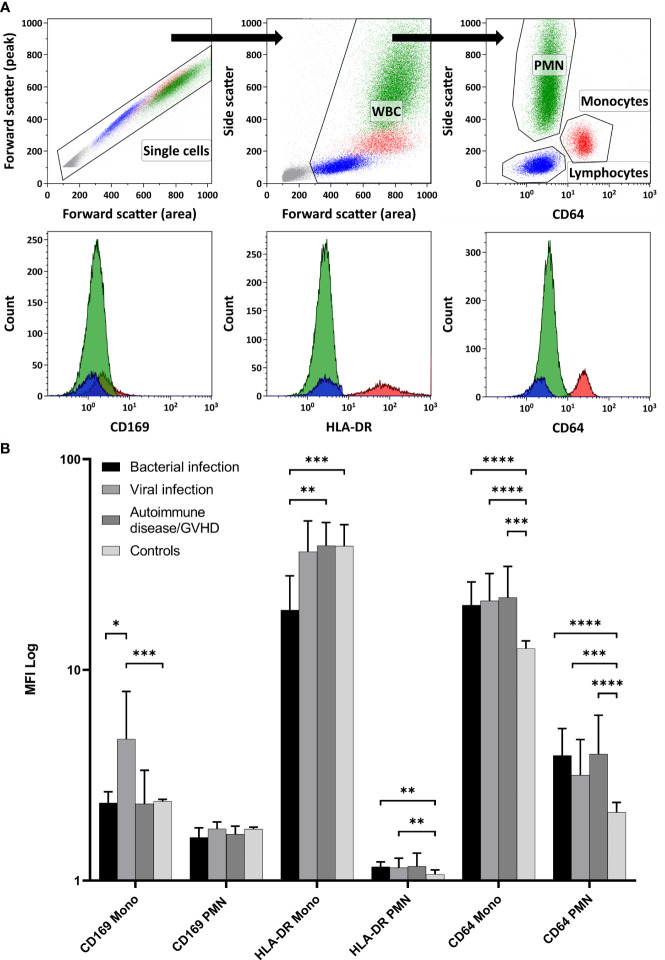
Expression of the Myeloid Activation Markers on Leukocytes from Patients with Inflammation. **(A)** demonstrates the flow cytometry gating strategy, along with representative histogram plots for polymorphonuclear neutrophils (PMN), monocytes, and lymphocytes (negative control population). In **(B)** CD169, HLA-DR, and CD64 expressions were quantified using relative MFI (Median and 95%CI) on PMN and monocytes (Mono). Controls displayed significantly lower CD64 expression in both PMN and monocytes than all other inflammatory states (Mann-Whitney test). Among the inflammatory conditions, CD169 showed higher expression on monocytes in viral infections than in bacterial infections. Furthermore, HLA-DR on monocytes was more pronounced in patients with autoimmune disease/GVHD than in patients with bacterial infections. (WBC, White blood cells; MFI, Median fluorescence intensity; GVHD, Graft versus host disease; 95%CI, 95% Confidence interval; * p < 0.05; ** p < 0.01; *** p < 0.001; **** p < 0.0001).

Relative MFI values from CD169, HLA-DR, and CD64 were estimated with flow cytometry on monocytes and PMN. The flow cytometry gating strategy is shown in **Figure 1A**. Relative MFI of CD64 was significantly higher in the patients with inflammation (bacterial infection, viral infection, and autoimmune disease/GVHD) than without inflammation (controls) either on monocytes or PMN (all p < 0.001; Mann-Whitney test) as shown in **Figure 1B**. Within the different inflammatory states, only CD169 was significantly higher expressed on monocytes in patients with viral infections than in patients with bacterial infection (p = 0.012), and HLA-DR on monocytes was higher in patients with autoimmune disease compared to patients with bacterial infections (p = 0.005) (**Figure 1**).

Mean values, along with their upper and lower 95% confidence intervals for the CRP and hemogram, are presented in **Table 2**. As expected, CRP values were significantly higher in bacterial infection compared to autoimmune disease/GVHD and controls (p < 0.0001 for both). However, they were not significantly different compared to viral infections (p = 0.061). The results of the Mann-Whitney U test, comparing all parameters presented in **Table 2**, are detailed in the Supporting Information (**Table S2**). Leveraging insights from the readily available N = 18 parameters (**Table 2** CRP/hemogram N = 12; **Figure 1B** MFI values: N = 6), we initially utilized the XAI system *C5.0* to distinguish controls from patients with inflammatory syndromes. The accuracy of this initial step was 97.46% (95%CI 92.75 ─ 99.47%). In the subsequent step, aiming to differentiate between bacterial infection, viral infection, and autoimmune disease/GVHD, the dataset was augmented in silico fourfold for each diagnosis using the SMOTE algorithm. Combining both steps yielded an overall accuracy of 81.0% (95%CI 72 – 87%). The classification of all N = 118 cases is presented in a confusion matrix in **Table 3**.

**Table 2:** CRP and Hemogram values.

	Bacterial Infection Mean (95%CI) N = 30	Viral Infection Mean (95%CI) N = 26	AID/GVHD Mean (95%CI) N = 24	Controls Mean (95%CI) N = 38
**CRP** **mg/L**	113.26 (72.22 - 154.30)	66.94 (34.54 - 99.35)	23.67 (10.06 - 37.27)	11.75 (3.89 - 19.62)
**WBC** **G/L**	8.95 (7.25 - 10.65)	8.10 (5.67 - 10.52)	7.37 (5.56 -9.17)	5.51 (5.01 - 6.02)
**Hemoglobin** **g/dL**	104.40 (96.21 - 112.59)	117.88 ( 108.07 - 127.69)	101.75 (89.95 - 113.55)	110.69 (100.41 - 120.98)
**Platelets** **G/L**	250.67 (189.70 - 311.63)	187.98 (144.19 - 231.77)	139.42 (86.99 - 191.85)	235.05 (198.70 - 271.40
**PMN** **%**	67.30 (58.22 - 76.38)	65.15 (54.69 - 75.61)	70.08 (61.93 - 78.23)	56.81 (52.62 - 61.00)
**PMN** **G/L**	6.80 (4.83 - 8.77)	5.81 (3.82 - 7.79)	5.30 (3.68 - 6.91)	3.21 (2.77 - 3.64)
**Eosinophils** **%**	1.18 (0.59 - 1.76)	0.74 (0.14 - 1.34)	2.94 (0.57 - 5.31)	1.92 (1.38 - 2.46)
**Eosinophils** **G/L**	0.08 (0.04 - 0.12)	0.05 (0.01 - 0.10)	0.26 (0.01 - 0.51)	0.10 (0.07 - 0.13)
**Basophils** **%**	0.56 (0.38 - 0.73)	0.33 (0.18 - 0.47)	0.29 (0.17 - 0.42)	0.55 (0.38 - 0.71)
**Basophils** **G/L**	0.05 (0.03 - 0.07)	0.03 (0.01 - 0.04)	0.02 (0.01 - 0.03)	0.03 (0.02 - 0.04)
**Monocytes** **%**	11.85 (8.66 - 15.05)	7.77 (5.68 - 9.86)	7.54 (5.74 - 9.34)	11.73 (9.82 - 13.63)
**Monocytes** **G/L**	0.92 (0.72 - 1.13)	0.50 (0.37 - 0.63)	0.75 (0.27 - 1.22)	0.63 (0.51 - 0.74)

CRP, C-reactive protein; WBC, White blood count; PMN, Polymorphonuclear neutrophils; CI, Confidence interval; AID, Autoimmune disease; GVHD, Graft versus host disease.

**Table 3:** Confusion Matrix for the Training- and Cross-Validation cohort.

		Prediction						
		Bacterial infection	Viral infection	AID/GVHD	Controls	Accuracy	Sensitivity	Specificity
**True diagnosis**	**Bacterial infection**	25	3	2	0	90.29%	86.21%	94.38%
	**Viral infection**	1	22	2	1	79.97%	64.71%	95.24%
	**AID/GVHD**	3	9	10	2	78.98%	71.43%	86.53%
	**Controls**	0	0	0	38	96.34%	92.68%	100%

AID, Autoimmune disease; GVHD, Graft versus host disease.

**Figure 2:**
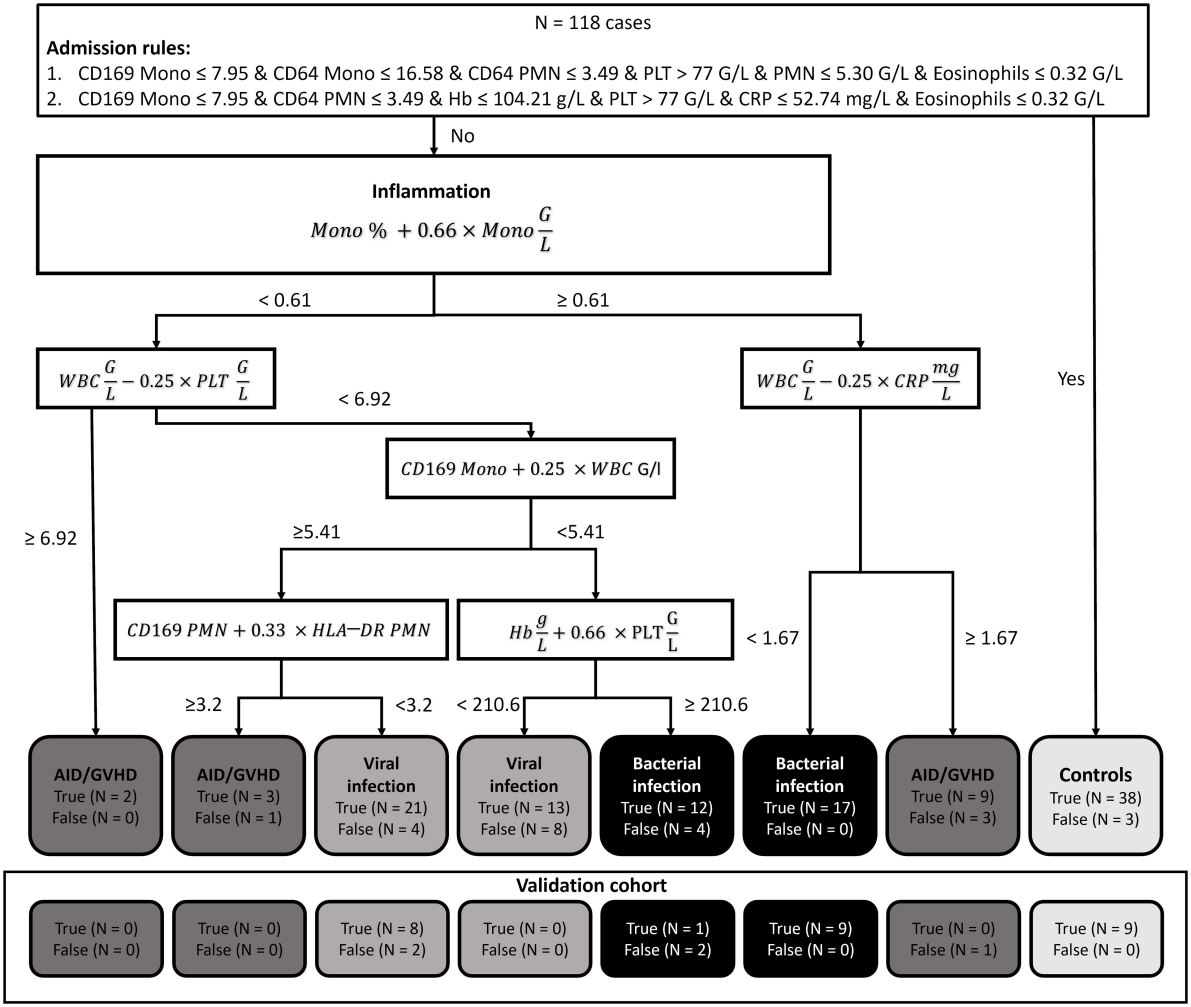
Explainable artificial intelligence (XAI) decision tree. In our diagnostic guidance approach, we initially employed two Boolean rules using the XAI system *C5.0* to distinguish between controls and patients presenting with inflammatory syndromes. Subsequently, we harnessed XAI to craft a bivariate binary decision tree. This tree, grounded on the Simpson impurity function, produced dichotomous rules based on basic arithmetic operations. These rules were designed to differentiate among bacterial infections, viral infections, and autoimmune diseases/graft-versus-host disease. (N, Number; Mono, Monocytes; PMN, Polymorphonuclear neutrophils; WBC, White blood count; Hb, Hemoglobin; CRP, C-reactive protein; PLT, platelets; AID, Autoimmune disease; GVHD, Graft versus host disease).

The highest accuracy among the subgroups was observed in patients with bacterial infection (90.29%), followed by viral infection (79.97%) and autoimmune disease/GVHD (78.98%). Sensitivity was also highest in bacterial infection (86.21%), succeeded by autoimmune disease/GVHD (71.43%) and viral infection (64.71%). Conversely, specificity was notably high for viral infection (95.24%) and bacterial infection (94.38%), compared to a lower 71.43% in patients with autoimmune disease. The diagnostic process of the two-step XAI is depicted in **Figure 2**. It enables the physician to understand which clinical parameters influenced the XAI’s diagnosis decision and to assess the validity of these decisions for each specific case. The decision-making rules defined by the XAI are based on formulas, which can be seamlessly integrated and automatically calculated within a laboratory information system that encompasses all 18 parameters.

The overall classification of inflammatory cases exhibited notable reliability. However, an exception arose with nine instances of autoimmune disease/GVHD, which were misclassified as viral infections. This misclassification adversely impacted the sensitivity for the viral infection category and overall performance for the autoimmune disease/GVHD category. Given the smaller sample sizes, autoimmune disease (N = 12) and GVHD (N = 12) were grouped into a single category due to their pathophysiological similarities. Of the nine misclassified cases, four were diagnosed with GVHD, while five had an autoimmune disease. This distribution suggests that there was no distinct bias within the category that could account for the observed misclassification.

### 3.2 Validation cohort

The generalizability of our diagnostic algorithm was further verified using an independent validation cohort of 32 patients. Due to challenges in distinguishing between autoimmune disease/GVHD and viral infections, patients with autoimmune disease/GVHD were excluded from this validation cohort. This cohort included 12 patients with bacterial infections, 8 patients with viral infections, and 12 control subjects without inflammation. The algorithm’s effectiveness in accurately categorizing these patients was evaluated, with detailed outcomes depicted in **Figure 2** (Validation Cohort). The overall accuracy of the diagnostic algorithm was determined to be 84.38% (95%CI 67.21% ─ 94.72%). The corresponding confusion matrix is provided in the Supporting Information (**Table S3**).

### 3.3 Evaluation of parameter significance

**Figure 3:**
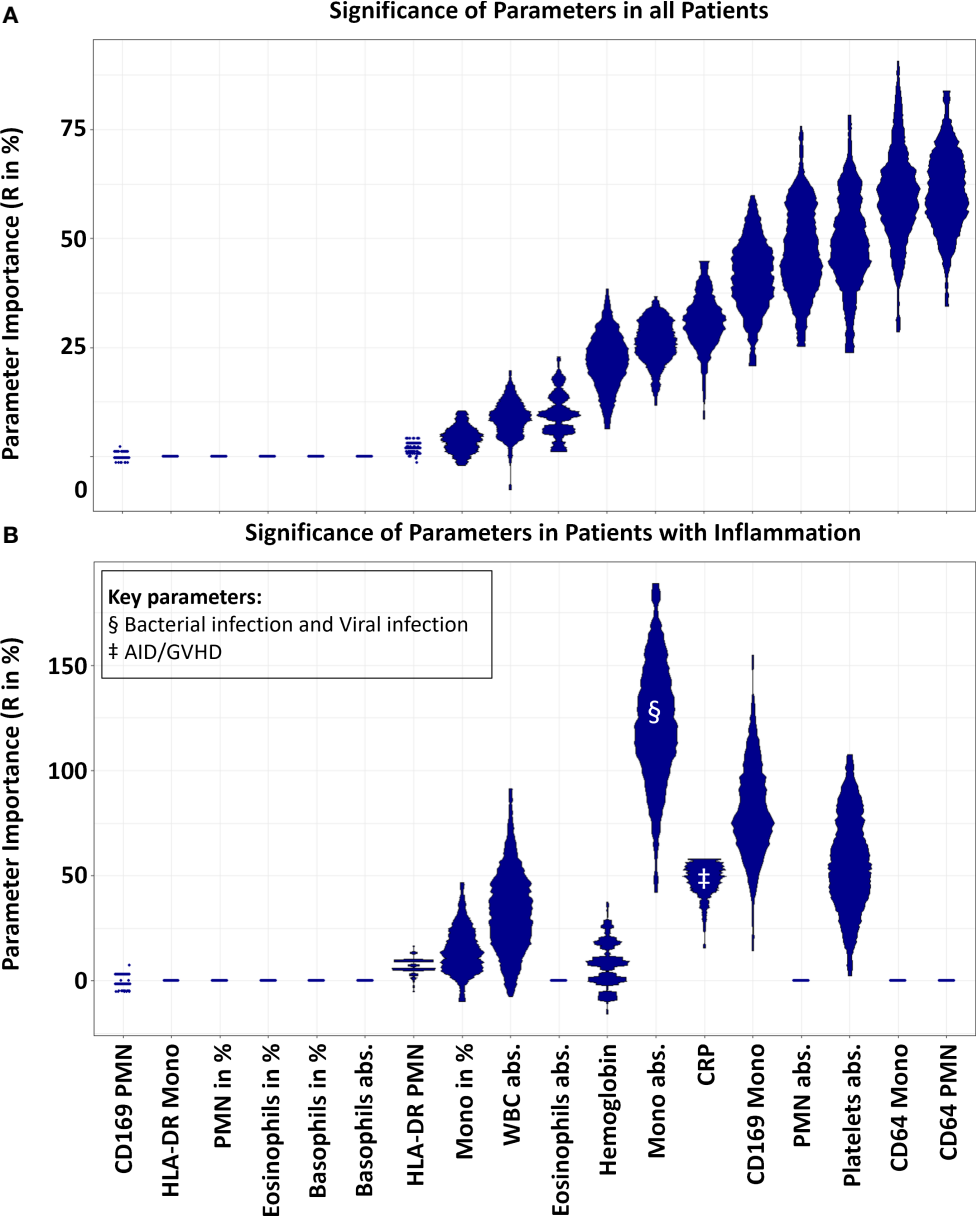
Parameter relevance for decision. In the mirrored-density plot, all 18 parameters were evaluated for their significance (R in %). A value of zero indicates that a feature is not relevant, while a high percentage signifies its high relevance. Panel **(A)** demonstrates the significance of the variables for all N = 118 samples of the main cohort, inclusive of 38 control cases without inflammatory disease. Here, CD64 expression on PMNs and monocytes emerged as the most crucial parameters. Conversely, Panel **(B)** highlights the importance (R in %) of the variables when differentiating solely between inflammatory states, excluding the control cases. In this context, the significance of CD64 expression on PMNs and monocytes reduced to zero, rendering them non-essential. Notably, the highest relevance was attributed to the absolute count of monocytes and the expression of CD169 on monocytes. In regard to the inflammatory subgroups, the monocyte count emerged as the key parameter for differentiating bacterial and viral infections, denoted by §. For distinguishing patients with autoimmune diseases/GVHD, CRP was identified as the key parameter, indicated by ‡. (Mono, Monocytes; PMN, Polymorphonuclear neutrophils; WBC, White blood count; CRP, C-reactive protein; AID, Autoimmune disease; GVHD, Graft versus host disease; R, Relevance).

We aimed to identify which of the 18 parameters compiled for each patient had the most significant influence on categorization by XAI and to determine if there were any parameters that could be considered redundant. To achieve this, we performed a procedure 200 times where we randomly permuted the values of a selected parameter (from the set of 18). Following each permutation, the predictions generated by the XAI were compared to our original classifications using the adjusted rand index as a metric. We then assessed the deviations of these results from our baseline prediction. In **Figure 3** MD-Plots illustrate the relevance (R in range (-)200 ─ 200%) for each parameter. This analysis provided insight into the extent to which introducing randomness impacted the precision of the XAI’s predictions.

The relative MFI of CD64 on PMN and monocytes emerged as a paramount parameter, influencing the XAI prediction with R-values of 61.21% and 59.70% respectively. In contrast, the results from the XAI prediction were minimally affected by the relative MFI of CD169 and HLA-DR (1.89%) on PMN (-0.34%) Moreover, the relative MFI of HLA-DR on monocytes appeared inconsequential, suggesting that the HLA-DR measure on myeloid cells was not a pivotal determinant in the prediction process. Additionally, the percentages of PMN, monocytes, and Eosinophils were substantially less influential (R = 0 ─ 3.65%) compared to their absolute values (R = 8.87 ─ 46.21%). Because N = 38 negative controls might have affected the relevance for all cases, we analysed the importance of the parameters to differentiate between the different inflammatory cases without controls. Results changed tremendously, and MFI of CD64 on PMN and monocytes turned out to decline from most relevant to irrelevant (R = 0% for both, **Figure 3B**). Most important was the count of the monocytes (R = 118.81%) followed by the CD169 MFI on monocytes (R = 79.47%).

## 4 Discussion

We established straightforward ruleset to aid in the differential diagnosis of inflammatory syndromes for physicians in the emergency department. These rules were derived from an XAI algorithm that utilized 18 readily available parameters from 80 patients with inflammation and 38 control subjects in a training- and cross-validation cohort. The overall accuracy reached 81% in the main cohort and increased to 84% in a smaller, independent validation cohort that excluded patients with AID/GVHD. We prioritized ensuring that our approach was both transferable and verifiable in low-income countries and in emergency departments worldwide. All outcomes from the 18 parameters can be ascertained within 2 hours. The XAI approach enables physicians to understand which clinical parameters, such as the monocyte count, were pivotal in the diagnostic decision-making. Consequently, a physician can evaluate whether the XAI’s reliance on specific parameters, like the monocyte count, is appropriate for their suspected diagnosis. This assessment allows for a critical determination of the XAI’s applicability to individual patient cases, especially in situations where certain parameters may be misleading for the physician’s differential diagnosis. By interacting with the XAI system and understanding its diagnostic processes, physicians can identify potential gaps in data. This interaction allows them to suggest additional clinically relevant parameters based on their medical expertise, which could enhance the performance of the XAI in diagnosing and guiding treatment decisions for patients with inflammatory conditions.

Notably, bacterial infections were identified with an accuracy of 90.29% (Sensitivity 86.21%; Specificity 94.38%). This accuracy can inform antibiotic treatment decisions, potentially reducing antibiotic overuse and the subsequent development of drug resistance. The accuracy for detecting viral infections stood at 79.97% (Sensitivity 64.71%; Specificity 95.24%). This suggests that when the guidelines do not indicate a viral infection, such an infection can be safely ruled out. However, a notable challenge arose with N = 9 patients with AID/GVHD being misclassified as having viral infections. This misclassification rate is significant, affecting 37.5% of AID/GVHD patients and 11.25% of all inflammatory cases, thereby impacting our overall findings. The established ruleset effectively distinguished control cases without inflammation. They reliably detect and rule out bacterial infections, and they exclude viral infections with a high degree of confidence. The primary challenge lies in diagnosing AID/GVHD. This may be attributed to the small sample size and the possibility that AID and GVHD differ considerably from each other, rendering the disease category too heterogeneous. It may be beneficial to consider additional markers to enhance accuracy in the AID/GVHD categorization. It is noteworthy that viral infections and GVHD are intricately related. Viral infections may precede or occur concurrently with GVHD (34–38). Furthermore, GVHD and its treatment can lead to impaired T-cell recovery, predisposing individuals to viral infections (39). Additionally, the clearance of viral infections is often delayed in highly immunocompromised individuals (38, 39). In our approach to differentiate various inflammatory conditions, we primarily relied on parameters involving myeloid cells. However, specifically for distinguishing between viral infections and autoimmune diseases, the integration of elements from the specific immune system, such as T-helper cell differentiation (Th1, Th17, Tregs), cytotoxic T-cells, and B-cells, might be beneficial. This additional focus on the specific immune system could enhance our ability to identify and differentiate these conditions accurately. Integrating the results from a basic lymphocyte subset analysis, which differentiates between T-helper cells, cytotoxic T-cells, NK-cells, and B-cells, could further refine our findings (40). This approach aligns with our strategy of utilizing easily accessible parameters.

We evaluated each parameter for its significance within the decision-making guidelines. Interestingly, CD64 expression on both monocytes and PMN proved crucial in ruling out non-inflammatory cases. However, within the scope of inflammatory cases, CD64 did not differentiate between bacterial infection, viral infection, and AID/GVHD. The expression of CD64 on PMN is a well-documented biomarker for the prediction of sepsis (7).

Nonetheless, interferon gamma (IFNγ), known to induce CD64 expression, plays a pivotal role in the immune regulation of viral and bacterial infections, cancer, and autoimmune diseases (9). As such, relying solely on CD64 expression may lead to misinterpretation of inflammatory states. Bourgoin et al. demonstrated that a combination of CD64 and CD169 (Siglec-1) expression on white blood cells could aid in differentiating between viral and bacterial infections (10–12).

These findings underscore the necessity of judiciously selecting the most appropriate control group tailored to the research question. The most significant parameters for distinguishing solely between inflammatory cases were the monocyte count and the CD169 expression on monocytes. Within our guidelines, parameters associated with monocytes played a pivotal role, particularly in the crucial step of differentiating inflammatory conditions. Yet, these findings might be uniquely relevant to our decision tree. In future studies, the set of 18 non-complementary parameters should be further refined and reduced.

Furthermore, absolute values appeared to provide more meaningful information within these guidelines and might be favoured over relative values in subsequent studies.

We synthesized cases to employ XAI algorithms in generating the bivariate decision tree for the second differentiation step of inflammatory states. It is argued that by using decision trees, this methodology doesn’t compromise the accuracy of the findings (22).

In conclusion, our study demonstrates that the application of XAI algorithms in differentiating various inflammatory conditions is promising, yet requires further refinement and expansion to enhance its accuracy and clinical applicability.

## Data availability statement

The raw data supporting the conclusions of this article will be made available by the authors, without undue reservation.

## Ethics statement

The studies involving humans were approved by Ethikkommission FB 20, Philipps-Universität Marburg, Marburg, Germany. The studies were conducted in accordance with the local legislation and institutional requirements. The participants provided their written informed consent to participate in this study.

## Author contributions

JH: Conceptualization, Methodology, Project administration, Writing – original draft. AR: Conceptualization, Investigation, Methodology, Writing – review & editing. AN: Funding acquisition, Supervision, Writing – review & editing. CB: Conceptualization, Supervision, Writing – review & editing. MT: Conceptualization, Formal analysis, Methodology, Software, Writing – original draft.
