# Supplementary material for: Development of an explainable AI system using routine clinical parameters for rapid differentiation of inflammatory conditions
Source: Front Immunol. 2024 Mar 6;15:1364954. doi: 10.3389/fimmu.2024.1364954 (PMC10950914; doi:10.3389/fimmu.2024.1364954)
Supplement: Supplementary file 1 [file DataSheet_1.pdf]

Supporting information

Table S1 Control Patients

**Initial Cohort**	**Number**
**Cancer Follow-up**	
- Acute Leukemia	8
- Lymphoma/Myeloma	10
- Pancreatobiliary cancer	6
- Gastroesophageal cancer	3
- Colorectal cancer	3
- Lung cancer	3
- Mamma cancer	2
- Sarcoma	1
**Healthy**	2
**Validation Cohort**	**Number**
**Cancer Follow-up**	
- Acute Leukemia	3
- Lymphoma/Myeloma	5
- Adrenocortical cancer	1
- Germ cell tumor	1
**Cholestasis**	1
**Aplastic Anemia (Follow-up)**	1

Table S2 Mann-Whitney U Test (Hemogram/CRP)

	P value	Mean rank of Bacterial Infection	Mean rank of Viral infection	Mean rank diff.	Mann-Whitney U
WBC G/L	0.179096	30.67	24.8	5.867	295
Hemoglobin g/dL	**0.038794**	23.93	32.88	-8.947	253
Platelets G/L	0.16055	30.78	24.66	6.123	291.5
CRP mg/L	0.060837	31.7	23.56	8.14	264
PMN %	0.893242	25.27	24.7	0.5736	292
PMN G/L	0.384075	26.69	23.09	3.605	255
Eosinophils %	0.205343	27.4	22.28	5.121	236.5
Eosinophils G/L	0.125074	27.9	21.72	6.186	223.5
Basophils %	**0.044447**	28.83	20.67	8.153	199.5
Basophils G/L	**0.037285**	28.94	20.54	8.399	196.5
Monocytes %	0.054378	28.08	20.27	7.804	193
Monocytes G/L	**0.001012**	30.46	17.45	13.01	131

	P value	Mean rank of Bacterial Infection	Mean rank of AID/GVHD	Mean rank diff.	Mann-Whitney U
WBC G/L	0.208783	29.93	24.46	5.475	287
Hemoglobin g/dL	0.669015	28.33	26.46	1.875	335
Platelets G/L	**0.016409**	32.07	21.79	10.28	223
CRP mg/L	**0.000023**	35.27	17.79	17.48	127
PMN %	0.800033	24.5	25.57	-1.065	286
PMN G/L	0.275422	27.12	22.61	4.507	244
Eosinophils %	0.804222	24.52	25.54	-1.024	286.5
Eosinophils G/L	0.644998	25.88	24	1.885	276
Basophils %	**0.04925**	28.75	20.76	7.989	201.5
Basophils G/L	**0.043801**	28.83	20.67	8.153	199.5
Monocytes %	**0.03324**	29.08	20.39	8.686	193
Monocytes G/L	**0.018353**	29.5	19.91	9.587	182

	P value	Mean rank of Bacterial Infection	Mean rank of Controls	Mean rank diff.	Mann-Whitney U
WBC G/L	**0.000029**	45.42	25.88	19.54	242.5
Hemoglobin g/dL	0.086541	29.87	38.16	-8.291	431
Platelets G/L	0.780129	35.27	33.89	1.372	547
CRP mg/L	**<0.000001**	48.83	21.97	26.86	110
PMN %	**0.001411**	41.33	26.46	14.87	264.5
PMN G/L	**0.000222**	42.62	25.58	17.04	231
Eosinophils %	**0.023701**	26.17	36.83	-10.66	329.5
Eosinophils G/L	0.099443	27.87	35.67	-7.806	373.5
Basophils %	0.730215	33.48	31.83	1.652	468.5
Basophils G/L	0.11482	36.87	29.51	7.352	380.5
Monocytes %	0.651684	31.21	33.38	-2.17	460.5
Monocytes G/L	**0.002879**	40.77	26.84	13.93	279

	P value	Mean rank of AID/GVHD	Mean rank of Viral infection	Mean rank diff.	Mann-Whitney U
WBC G/L	0.8313	24.54	25.44	-0.8983	289
Hemoglobin g/dL	0.066103	21.17	28.68	-7.513	208
Platelets G/L	0.125532	21.79	28.08	-6.288	223
CRP mg/L	**0.018569**	20.13	29.68	-9.555	183
PMN %	0.699411	24.28	22.72	1.565	246.5
PMN G/L	0.917805	23.28	23.72	-0.4348	259.5
Eosinophils %	0.28164	25.59	21.41	4.174	216.5
Eosinophils G/L	0.420426	25.07	21.93	3.13	228.5
Basophils %	0.778512	22.93	24.07	-1.13	251.5
Basophils G/L	0.819335	23.96	23.04	0.913	254
Monocytes %	0.906049	23.24	22.75	0.4891	247.5
Monocytes G/L	0.879372	23.3	22.68	0.6225	246

	P value	Mean rank of Controls	Mean rank of Viral infection	Mean rank diff.	Mann-Whitney U
WBC G/L	0.060859	28.49	37.34	-8.853	341.5
Hemoglobin g/dL	0.652705	31.14	33.3	-2.155	442.5
Platelets G/L	0.140053	34.78	27.78	6.996	369.5
CRP mg/L	**0.000005**	23.28	43.66	-20.38	158.5
PMN %	**0.0096**	26.46	38.5	-12.04	264.5
PMN G/L	**0.038247**	27.34	37.04	-9.701	298
Eosinophils %	**0.000101**	37.63	20.04	17.59	185
Eosinophils G/L	**0.000399**	37.08	20.96	16.12	206
Basophils %	**0.027266**	34.87	24.61	10.26	290
Basophils G/L	0.066113	34.17	25.76	8.41	316.5
Monocytes %	**0.001337**	35.89	21.18	14.71	213
Monocytes G/L	0.108406	33.26	25.73	7.536	313

	P value	Mean rank of AID/GVHD	Mean rank of Controls	Mean rank diff.	Mann-Whitney U
WBC G/L	0.263353	34.75	29.45	5.303	378
Hemoglobin g/dL	0.134456	27.17	34.24	-7.07	352
Platelets G/L	**0.003221**	23.13	36.79	-13.66	255
CRP mg/L	**0.029488**	37.13	27.03	10.1	297
PMN %	**0.000706**	40.67	25.14	15.53	214.5
PMN G/L	**0.016959**	37.93	26.8	11.13	277.5
Eosinophils %	0.116564	26.41	33.78	-7.363	331.5
Eosinophils G/L	0.056839	25.46	34.36	-8.899	309.5
Basophils %	**0.018462**	24.2	35.12	-10.92	280.5
Basophils G/L	0.267331	27.8	32.93	-5.13	363.5

Monocytes %	**0.002768**	22.39	36.21	-13.82	239
Monocytes G/L	0.356973	28.28	32.64	-4.362	374.5

Table S3 Confusion matrix Validation cohort

		Prediction						
		Bacteri alinfecti on	Viral infectio n	AID/ GVHD	Control s	Accu-racy	Sensi-tivity	Speci-ficity
True diagnosis	Bacterial infection	10	2	0	0	86.67%	83.33%	90.00%
	Viral infection	0	8	0	0	95.83%	100%	91.67%
	AID/GVHD	0	0	0	0	NA	NA	NA
	Controls	2	0	1	9	87.50%	75.00%	100%
